# Spatial subsidies in spider diets vary with shoreline structure: Complementary evidence from molecular diet analysis and stable isotopes

**DOI:** 10.1002/ece3.2536

**Published:** 2016-10-26

**Authors:** Peter A. Hambäck, Elisabeth Weingartner, Love Dalén, Helena Wirta, Tomas Roslin

**Affiliations:** ^1^ Department of Ecology, Environment and Plants SciencesStockholm UniversityStockholmSweden; ^2^ Department of Bioinformatics and GeneticsSwedish Museum of Natural HistoryStockholmSweden; ^3^ Department of Agricultural SciencesUniversity of HelsinkiHelsinkiFinland; ^4^ Department of EcologySwedish University of Agricultural SciencesUppsalaSweden

**Keywords:** Baltic Sea, chironomids, DNA barcoding, *Pardosa*, stable isotope analysis

## Abstract

Inflow of matter and organisms may strongly affect the local density and diversity of organisms. This effect is particularly evident on shores where organisms with aquatic larval stages enter the terrestrial food web. The identities of such trophic links are not easily estimated as spiders, a dominant group of shoreline predator, have external digestion. We compared trophic links and the prey diversity of spiders on different shore types along the Baltic Sea: on open shores and on shores with a reed belt bordering the water. A priori, we hypothesized that the physical structure of the shoreline reduces the flow between ecosystem and the subsidies across the sea–land interface. To circumvent the lack of morphologically detectable remains of spider prey, we used a combination of stable isotope and molecular gut content analyses. The two tools used for diet analysis revealed complementary information on spider diets. The stable isotope analysis indicated that spiders on open shores had a marine signal of carbon isotopes, while spiders on reedy shores had a terrestrial signal. The molecular analysis revealed a diverse array of dipteran and lepidopteran prey, where spiders on open and reedy shores shared a similar diet with a comparable proportion of chironomids, the larvae of which live in the marine system. Comparing the methods suggests that differences in isotope composition of the two spider groups occurred because of differences in the chironomid diets: as larvae, chironomids of reedy shores likely fed on terrestrial detritus and acquired a terrestrial isotope signature, while chironomids of open shores utilized an algal diet and acquired a marine isotope signature. Our results illustrate how different methods of diet reconstruction may shed light on complementary aspects of nutrient transfer. Overall, they reveal that reed belts can reduce connectivity between habitats, but also function as a source of food for predators.

## 1 Introduction

In heterogeneous landscapes, the proximity of productive habitats often has strong effects on both growth and abundance of species in adjacent habitats (Polis & Hurd, 1996). Not surprisingly, subsidies of various types (i.e., inflows of detritus, nutrients, or organisms) between neighboring habitats have attracted much interest in ecology (Birkhofer, Wise, & Scheu, 2008; Hilderbrand, Hanley, Robbins, & Schwartz, 1999; Nakano & Murakami, 2001; Nowlin, Vanni, & Yang, 2008; Sanzone et al., 2003). The focal point of these studies is typically the extent to which local productivity at different trophic levels, population dynamics, and community structure is affected not only by processes and energy accumulation in the local habitat but also by the inflow of energy and biomass from other habitats (Anderson, Wait, & Stapp, 2008; Hocking & Reynolds, 2011; Polis, Anderson, & Holt, 1997; Sabo & Power, 2002). For instance, Leroux and Loreau (2008) argued that low‐lying ecosystems may experience larger spatial subsidies because matter tends to flow downhill. In addition, it is known that the structure of the transition zone between habitats may affect the size of the inflow by increasing or decreasing habitat connectivity (Delettre & Morvan, 2000; Polis et al., 1997), but also because the transition zone between ecosystems may be highly productive by itself.

The flow of matter and energy between aquatic and terrestrial systems is one example of spatial subsidies that has received considerable interest (Bartels et al., 2012; Dreyer, Hoekman, & Gratton, 2012; Hoekman, Dreyer, Jackson, Townsend, & Gratton, 2011; Kolb, Jerling, & Hambäck, 2010; Stapp & Polis, 2003). Nutrient‐rich wetlands, lakes, and marine areas often provide productive habitats not only for aquatic but also for terrestrial species, especially for generalist arthropod predators that consume insects emerging from the water (Collier, Bury, & Gibbs, 2002; Hodkinsson, 1999; Paetzold, Lee, & Post, 2008). Vertebrate predators such as bats or lizards and invertebrate predators such as spiders often preferentially feed on midges and other insects with aquatic larval stages (Mellbrand & Hambäck, 2010; Paetzold, Smith, Warren, & Maltby, 2011; Rydell, 1989; Sabo & Power, 2002; Swift & Racey, 1983). Not surprisingly, densities of these predators are typically higher close to various water bodies and depend on the amount of inflow of prey on the shoreline (Dreyer et al., 2012; Iwata, 2007; Laeser, Baxter, & Fausch, 2005; Mellbrand, Östman, & Hambäck, 2010; Paetzold et al., 2011). Spiders in particular are very abundant on shorelines, where they may attain high densities and spider webs may border the water wherever structures to anchor the webs are provided.

Despite this apparent effect on spider densities, there are few direct estimates of spider usage of aquatic prey items (but see Henschel, 2004 for an example with web spiders). An important reason for this lack of data is the external digestion employed by spiders, which leaves few traces of prey remains in the spider gut. Traditional, morphological‐based gut analysis is therefore not possible and spider diets are typically inferred indirectly using stable isotope analyses (SIA) (Marczak & Richardson, 2007; Sanzone et al., 2003; Wise, Moldenhauer, & Halaj, 2006). SIA is particularly useful at marine shorelines due to the very different stable isotope composition of marine and terrestrial prey (e.g. Paetzold et al., 2008), caused by the different isotope ratios in marine and terrestrial plants. Using SIA, recent analyses show that wolf spiders on marine shorelines have a high proportion of marine carbon incorporated in their body, suggesting a diet either of insects with marine larvae and terrestrial adults (such as chironomids) or of insects that feed directly or indirectly on marine detritus on the shoreline (Mellbrand & Hambäck, 2010; Mellbrand, Lavery, Hyndes, & Hambäck, 2011).

The ability to perform gut content analysis of spiders has improved dramatically in recent years with the technical developments on DNA sequencing, which has expanded the toolbox for ecologists interested in trophic interactions. Gut content analyses using antibodies or species‐specific primers have been used for some time (Chapman, Schmidt, Welch, & Harwood, 2013; Kuusk & Agusti, 2008; Virant‐Doberlet, King, Polajnar, & Symondson, 2011), but they require that the potential prey species are already known. Sequencing of gut contents without detailed knowledge of potential prey has a more recent history (Clare, 2014) and has only recently been used for spider guts (Piñol, San Andres, Clare, Mir, & Symondson, 2014; Wirta et al., 2015b). This technique allows species‐level analyses, but then relies on access to a well‐populated reference library of taxon‐specific sequences. What these analyses will not reveal is the origin of the prey, as they attribute samples to taxa, not to habitats. DNA‐based techniques have also been criticized for a lack of quantitative information, as any biases in DNA extraction or amplification will essentially propagate to and accumulate in downstream analyses (Clare, 2014; Deagle, Thomas, Shaffer, Trites, & Jarman, 2013; Piñol et al., 2014; Pompanon et al., 2012).

In the Baltic Sea, and elsewhere, shores differ in structure, potentially causing differences both in the predator community and in the strength of the cross‐ecosystem flow of organisms. In our study area, one structural difference is between open shores, where spiders hunt for prey in grass close to the shore, and reedy shores, where spiders rather move inside the reed belt. Because reed belts can be fairly tall, they may reduce movements of chironomids and other emerging insects from the marine to the terrestrial system. We would thereby expect spiders on reedy shores to have a lower proportion of chironomids in their diet compared to spiders on open shores. Nonetheless, the reed belts may also by themselves contain high densities of various spider prey, such as dipterans feeding in the reed belts.

In this study, we combine diet analyses using SIA and molecular gut content analyses to study the trophic niche of dominant shoreline wolf spiders in habitats directly adjacent to the Baltic Sea and in habitats that are separated from the water by a reed belt. Our objectives were fourfold: (1) to study the effect of reed belts on the connections between the marine and terrestrial habitats; (2) to detect the actual links (taxa) for the marine–terrestrial transfer; (3) to compare the niches of spider taxa; (4) to test the ability of our DNA‐based technique in providing quantitative information on predation rates. The results suggest that the two methods provided complementary information on spider diets and on the different roles of marine inflow on open and reedy shores.

## 2 Methods

### 2.1 Field sampling

**Figure 1:**
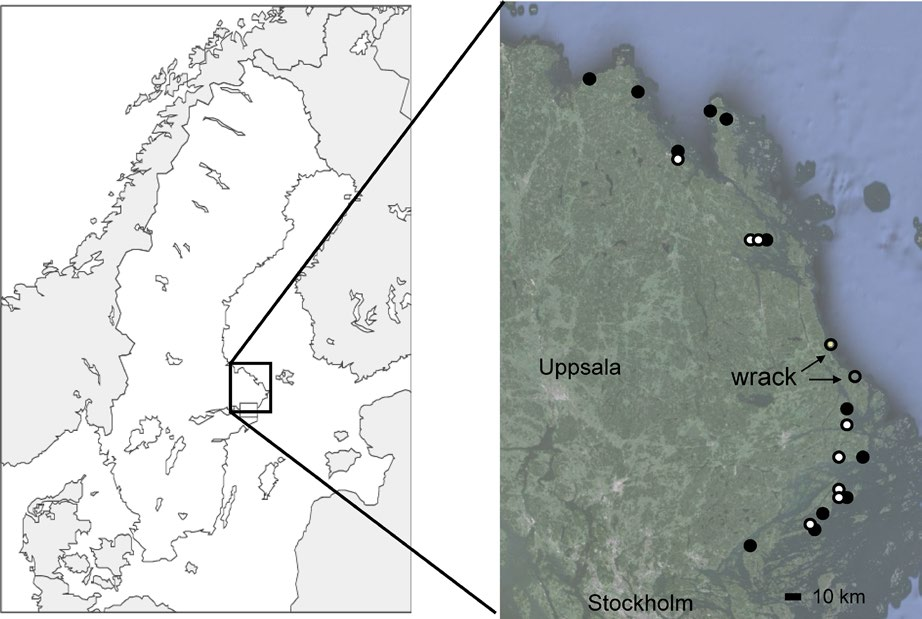
Map of sampling sites, in Uppland county north of Stockholm, Sweden (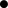 = open shores, 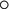 = reedy shores). Satellite imagery © 2012 DigitalGlobe, imagery date: 13/12/2015, Google Earth

Spiders were collected from 20 sites along the Baltic Sea coast just north of Stockholm, Sweden (Figure 1). Sites were selected to include both open shores adjacent to the water and areas close to the water but with a reed belt adjacent and into the water. The vegetation on open shores was scant, with interspersed stony areas. The reedy shores had a more uniform vegetation cover typical of coastal marshes, with grasses, sedges, and forbs. Due to the reed belt, these sites were also farther away from the open water than the open shore sites. Our intention was to also include sites with heavy wrack deposition, but we were only able to locate two sites in the area. Because this limited sample still showed some interesting patterns, we include the data but note that the comparison with wrack spiders is weak.

On each site, we collected at least 50 wolf spiders within 30 m from the shore by hand during June when adults are active. Our intention was to get a representative sample of the dominant wolf spiders. The identification of species suggested that the sample was heavily dominated (>95%) by two species (*Pardosa amentata* and *Pardosa prativaga*), and other species were therefore excluded from further analysis. These two dominant species have also previously been shown to dominate Baltic Sea shorelines, and both species are known to use marine inflow to a large extent (Mellbrand & Hambäck, 2010), even though the use of marine inflow likely varies across the season depending on prey life cycles. The important marine prey (chironomids) in this system emerges across the whole summer, and can therefore be used by spiders in all seasons, but the species composition generally varies with the season (Egan, Ferrington, Lafrancois, & Edlund, 2015; Raunio & Paasivirta, 2008). In a previous study, Mellbrand and Hambäck (2010) showed that more than 75% of all cursorial predators on Baltic Sea shorelines, similar to the ones in this study, are spiders. In that study, more than 50% of all spiders represented a single species, *P. amentata*. In our study, *P. amentata* was captured only on open shores, while *P. prativaga* was captured to an equal extent on open and reedy shores. In addition to spiders, we also collected insects in pan traps. The intention was to complement DNA barcode libraries with potential prey species that occur in these particular sites.

All captured spiders and prey insects were transferred to individual tubes with 95% ethanol and stored in −20°C. Spiders were morphologically identified to species prior to further processing, whereas captured prey insects were first DNA‐barcoded and only identified morphologically when the DNA sequences had been found to match sequences in spider guts. After identification, the same spider individuals were used for three analyses. Spider legs (about 2 mg) were used for stable isotope analysis of both δ^15^N and δ^13^C. However, for the current purpose of identifying prey with a terrestrial versus marine resource base, we focus on δ^13^C. The spider abdomen (opisthosoma) was carefully halved, and the two halves were used in molecular analysis using two sequencing methods. In spiders, the gut is generally distributed between the prosoma and the abdomen with small extensions into the legs (Foelix, 1996).

### 2.2 Stable isotope analysis

For SIA, we explicitly used spider legs, as isotope signatures in these appendages have a longer turnover time (tissue half‐life ≈ 18 days) than those of the abdomen (tissue half‐life ≈ 8 days) (Belivanov & Hambäck, 2015). Thus, SIA of spider legs reflects the diet composition of spiders over a longer time period than the abdomen. Prior to analysis, spider legs for 10 individuals per site (five of each species) were freeze‐dried for at least 16 hr, weighed to the nearest 0.1 mg, and placed in small tin cups. Stable isotope ratios were measured using a PDZ Europa ANCA‐GSL elemental analyzer interfaced to a PDZ Europa 20‐20 isotope ratio mass spectrometer at UC Davis Stable Isotope facility (Davis, CA, USA). Isotope ratios were calculated as deviations from the international limestone standard Vienna PeeDee Belemnite (V‐PDB) (δ^13^C) in parts per thousand (‰): X = [(R_sample_/R_standard_) − 1] × 1,000, where X is the heavier isotope of the element (^13^C) and R is the isotopic ratio (^13^C/^12^C).

For comparison of carbon stable isotopes of prey and predators, we used data of collected plant material and dipteran prey from the same region on open and reedy shores (Enskog, 2006). Carbon isotope ratios from green algae, which is the most likely food source of marine chironomids, vary with salinity but for algae close to the study sites δ^13^C = −20.6‰ ± 3.5 (mean ± *SD*, N = 59). Terrestrial plants also vary in carbon isotope ratios between species, but for reeds δ^13^C = −26.6‰ ± 1.1 (N = 23) which is generally enriched compared to other terrestrial plants (δ^13^C = −30.3‰ ± 1.3, N = 128). Chironomids collected on open shores have δ^13^C = −18.4‰ ± 2.0 (N = 41) but for chironomids from reedy shores we lack specific data. For other Diptera (brachyceran flies) collected in reeds, δ^13^C = −25.6‰ ± 0.7 (N = 51). Unfortunately, we have no data on chironomids from reedy shores, because we assumed that chironomids on these shores would also have a marine origin and therefore that spiders feeding on chironomids also on reedy shores would have a marine carbon isotope signal.

### 2.3 Genetic analysis

To detect prey DNA in gut contents, we analyzed 20 individuals per species and site. In a few cases, samples were smaller but diets were always calculated as proportions. For the analysis, we used primers amplifying a 332‐bp‐long DNA fragment from Diptera and Lepidoptera and some other potential prey groups (Heteroptera, Coleoptera), but not from the spider itself. Two methods (Sanger sequencing and massive parallel sequencing) were used for the molecular gut content analysis of spiders for each half abdomen separately, because an initial objective was to test among methods. During the process, we found both methods to work well but they also provided partly nonoverlapping information on the spider diets. The cause for these differences is unclear but may be caused by differences in the DNA extraction or in primer satiation. For this reason, we decided to pool the data in the diet analysis. The complete laboratory procedure for the two methods, including PCR protocols, is described in Wirta, Weingartner, Hambäck, and Roslin (2015a) and is briefly described here.

In the first method, DNA was extracted from one‐half of the spider abdomens, amplified with primers LCO1490 and MlepR1 (Folmer, Black, Hoeh, Lutz, & Vrijenhoek, 1994; Hajibabaei, Janzen, Burns, Hallwachs, & Hebert, 2006; Rougerie et al., 2011) and amplicons were then directly sequenced by Sanger sequencing. In the second method, we pooled halves of up to 20 spider abdomens (within site and species) before extracting DNA twice. DNA extracts were amplified with tagged Diptera–Lepidoptera‐specific primers (same primers as above), using two separate tags for each DNA extraction. Thus, for every pool of spiders, there were four PCR products. The PCR products were cleaned and sequenced on a GS Junior (Roche 454) at the Molecular Systematics Laboratory of the Natural History Museum in Stockholm (for details on protocol see Wirta et al., 2015a).

The whole run (which also included a different data set) resulted in 125,565 sequences that passed the filter. The sequences were sorted per primer combination; adaptors and tags were removed using a perl script by Johan Nylander (BILS, SciLife Laboratories, Stockholm, Sweden). The sequences were further trimmed using Tagcleaner available at edwards.sdsu.edu/cgi‐bin/tagcleaner/tc.cgi. Primers were removed, and sequences shorter than 280 were discarded. Sequences were further trimmed in Mothur following the protocol 454 SOP (available at www.mothur.org, Schloss, Gevers, & Westcott, 2011). Sequences were trimmed to minimum length of 300 bp and maximum length of 311 bp, aligned against the data set of Sanger‐sequenced Diptera of 307 bp, screened to remove sequences shorter than 300 bp and filtered. Some prey species identified with method 2 were represented by only one to two sequences. We retained these singletons and doubletons in the analysis, but also note that the removal of them did not change results. In total, 47,663 sequences remained for the present analysis.

The derived gene sequences were compared to DNA barcodes in the Barcode of Life Database (BOLD, www.boldsystems.org, Ratnasingham & Hebert, 2007), using the identification engine relying on matches above 97%. As the target sequences were only 307 bp and as BOLD is far from complete as a database, identities should be treated with care. In the process of identification, we encountered several problems which we resolved differently. First, in some cases, queries returned a 100% match with North American species not occurring in the study area. In all these cases, species from the same genus do occur in the sampled area but lack sequences in the database. Second, in other cases, multiple species in the database yielded equally good matches, due to limited variability in the short target sequence. In most of these cases, knowledge about species distributions rendered one or several of these species less likely. This was, however, not the case for *Lygus* sp. (Heteroptera: Miridae), where seven species showed an equally likely match to the observed gene sequence. These sequences were treated only at the genus level, and multiple matches are reported. Third, sequences in the database sometimes derived from voucher individuals that had only been identified to genus or family. In these cases, we adopted the higher level taxonomic identity. Fourth, some sequences yielded no match exceeding the cutoff point of 97%. These sequences were blasted against Genbank. As this step was less precise, identities are then only reported at the family level. Fifth, all sequences from Chironomidae and Ceratopogonidae were blasted against unpublished data collected in the region by Thomas Lyrholm (Chironomidae) and Jonas Strandberg (Ceratopogonidae) at the Natural History Museum in Stockholm. Finally, as an independent control of the molecularly based identification procedure, we compared the performance of BOLD with morphological identification on 104 dipterans that were also morphologically identified.

In all cases where BOLD found a match >97%, this was confirmed with morphological identification at least to genus level and mostly to species level. For one case where a sequence abundantly detected in the gut content analysis was missing from the BOLD database, we were fortunate enough to detect a specimen in our separate pan trappings for DNA barcoding of potential prey. This specimen was later identified as a black scavenger fly (*Thripomorpha verralli* (Edw.), Scatopsidae) by Jean‐Paul Haenni. Overall, almost all sequences found in the spider guts could be resolved to either species, genus, or in some cases family. The latter was true for some Diptera groups, such as Anthomyidae, Sphaeroceridae, Phoridae, and Ceratopogonidae, which are hard to identify or where only a limited number of sequences are available in BOLD.

When taxon identification was completed, we tabulated the minimum number of predation events for each site and spider species from both methods combined. A predation event is defined as a case when we have unequivocal evidence from the molecular gut content analysis that a predator has consumed at least one individual of a prey species. Our ability to detect predation events differ between the methods. For method 1 (Sanger sequencing), each spider individual would yield a maximum of one prey sequence. Hence, every prey sequence detected was counted as a separate predation event and multiple predation events of the same prey species (in different spider individuals) could accordingly be recorded from the same spider species in the same site. However, multiple prey individuals of the same species consumed by the same spider individual could only be scored as a single predation event. For method 2 (as based on pooled samples), predation events were only recorded once for each combination of site, prey, and spider species, irrespective of the number of sequences—and only when the same taxon had not been recorded previously by Sanger sequencing. The logic is that the pooling of spider individuals precluded quantitative information on the number of predation events. For each site and spider species, we then calculated the proportional use for prey groups where the total number of recorded predation events exceeded ten: Ceratopogonidae, Chironomidae, Dolichopodidae, Ephydridae, Sphaeroceridae, and Lepidoptera. Our approach likely underestimated the number of predation events, not only because of the pooling of spider individuals but also because some prey taxa may have failed to amplify using the selected primers.

### 2.4 Statistics

For the comparison of stable isotope compositions among sites and species, we performed two analyses. First, we compared carbon isotope values for spiders from open and reedy shores. For this analysis, we calculated a mean isotope value for each site and species. Second, to compare niches among species, we focused on open shore sites where *P. amentata* and *P. prativaga* co‐occurred. Individual isotope values were fitted to a linear mixed‐effects model using site as random effect and spider species as fixed effect.

For the gut content analysis, we first compared the use of the six dominant prey groups (Ceratopogonidae, Chironomidae, Dolichopodidae, Ephydridae, Sphaeroceridae, and Lepidoptera) using the adonis function in the vegan package (Oksanen et al., 2016) among spider species (*P. amentata, P. prativaga*) and shore types (open shore, reedy shores, wrack). In order to identify the cause for significant variation exposed by the first model, we compared group pairs again using adonis. When group differences had been identified, we used indicator species analysis using the indval function in the labdsv package (Roberts, 2016) to identify the prey groups that differed among significant groups in the adonis analysis. Finally, we compared carbon stable isotopes with the proportional use of chironomids (as representing the likely marine inflow) using regression analysis as applied separately to open and reedy shores. Separate regressions were performed for the two shore types but combined between spider species because a visual inspection suggested different patterns between groups but not between species. One data point for open shores (*P. prativaga*) had a high leverage on the predicted relationship, but strongly resembled those of reedy shores. We therefore performed the analyses without this point, and return to a biological interpretation in the discussion.

## 3 Results

### 3.1 Stable isotope analysis

The SIA showed that spiders collected on open shores were more enriched in ^13^C (δ^13^C ± SE = −21.5‰ ± 0.3, N = 19) than spiders collected on reedy shores (δ^13^C = −26.2‰ ± 0.3, N = 8) (*F* = 70.2, *p *« .001), suggesting more marine carbon in spider legs from open than from reedy shores. When comparing δ^13^C between the two spider species *P. amentata* and *P. prativaga* on sites where both species occurred, we found that *P. amentata* (δ^13^C = −21.2‰ ± 0.5) was enriched in ^13^C compared to *P. prativaga* (δ^13^C = −22.6‰ ± 0.5) (Log ratio = 30.3, Δ*df* = 1, *p *< .0001, number of groups = 8), suggesting more marine carbon in *P. amentata* than in *P. prativaga* (recall: reed δ^13^C = −26.6‰ and green algae δ^13^C = −20.6‰, (Enskog, 2006)).

### 3.2 Gut content analysis

From 542 spider individuals, we recorded a total of 223 independent predation events (41% success rate representing 105 taxa, Appendix S1). Method 1 (Sanger sequencing) yielded 132 predation events, whereas the remainder of 92 events were added from method 2, as yielding a total 47,663 sequences. Among those sequences, 444 were identified as fungal sequences and were discarded. The resultant list of prey species was heavily dominated by Diptera (86 species, 203 predation events), followed by Lepidoptera (13 species, 14 predation events), Coleoptera (three species, three predation events), and Heteroptera (two species, three predation events).

**Figure 2:**
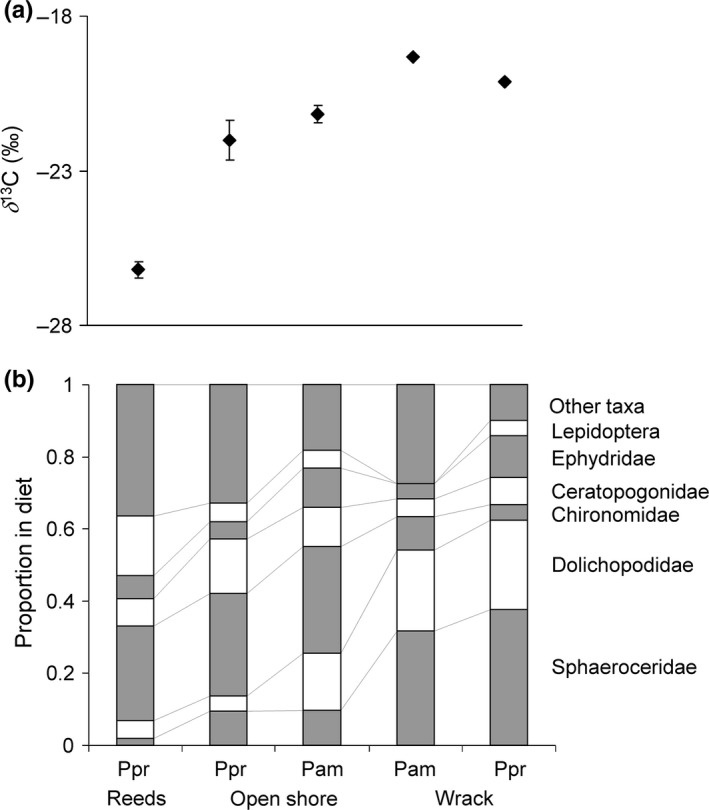
The carbon isotope composition (a) and the mean proportion of prey in the diets from the molecular gut content analysis (b) for *Pardosa prativaga* (Ppr) and *Pardosa amentata* (Pam) on reedy shores, open shores, and on shores with wrack. The order of prey taxa is identical in all groups, with the identity shown on the rightmost group

When comparing diets with adonis, we found an effect of shore type (*F*_2,27 _= 3.1, *p *< .004) but no effect of spider species (*F*_1,27 _= 1.7, *p *= .15). When comparing diets among shore types, we found differences between open shores and wrack (*F*_1,27 _= 5.4, *p *< .004), between reedy shores and wrack (*F*_1,10 _= 4.9, *p *< .007), but not between open and reedy shores (*F*_1,25 _= 1.4, *p *= .27). When comparing diets for spiders among shores using indicator species analysis, we find two groups that were more consumed on wrack compared to the other shore types: Sphaeroceridae (*p *< .04) and Dolichopodidae (*p *< .004). Relative abundances of prey types for each species and shore type are found in Figure 2.

### 3.3 Comparing carbon stable isotopes with the proportional use of chironomids

**Figure 3:**
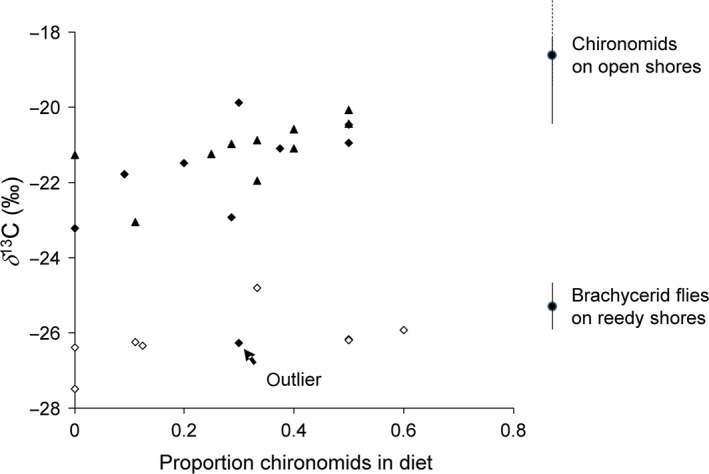
Relationship between the proportion of chironomids in spider guts, relative to the total number of predation event as revealed by molecular gut content analysis, and the stable isotope composition of spider legs (diamonds = *Pardosa prativaga*, triangles = *Pardosa amentata*, filled symbols = open shores, unfilled symbols = reedy shores). An outlier that was excluded from analysis is indicated, see text for justification. The dotted line shows the estimated relationship on open shores. For comparison, the stable isotope composition of chironomids collected on open shores and brachycerid flies from reedy shores is included (data from Enskog, 2006)

For open shores (Figure 3), we found that the proportion of chironomids explained 40% of the variation in δ^13^C (*F*_1,16 _= 12.2, *p *< .005, R^2 ^= 0.40). The estimated relationship was δ^13^C = −22.5 (SE = 0.4) + 3.9 (SE = 1.1) × proportion chironomids. This estimated relationship would suggest that δ^13^C = −22.5 in spider legs corresponds to a diet with no chironomids, while δ^13^C = −18.6 would correspond to a diet with only chironomids. For reedy shores (Figure 3), the relationship between the proportion of chironomids and δ^13^C was nonsignificant (δ^13^C = −26.6 (SE = 0.4), *F*_1,7_ = 2.2, *p* = .18).

## 4 Discussion

Spiders are no doubt important predators on marine, river, and lake shorelines. The dependence of spiders on aquatic prey has often been implied, and is perhaps obvious when examining the contents of spider webs situated close to the water. However, the data for a major group of shoreline spiders, wolf spiders, have been indirect, as derived from the composition of stable isotopes in spider bodies. While such data offer convincing evidence of the role of aquatic prey in spider diets, they fail to expose the detailed routes of carbon in food webs. In this study, we verified that spiders living on shores consume a large proportion of chironomids, a major insect group characterized by aquatic larvae and terrestrial adult stages. We also found that the proportion of chironomids in the diet is well correlated to the stable isotope composition of spider legs, suggesting that chironomids may indeed account for the signature of marine carbon isotope in shoreline spiders. However, the correlation between the stable isotope composition in spider legs and the proportion of chironomids was only apparent on open shores and not on reedy shores.

When comparing spider diets and dependence on marine production between open and reedy shores, firm conclusions were only made possible by the combination of SIA and gut content analysis. SIA indicated a distinct difference between spiders on open and reedy shores, where the former showed a carbon isotope composition characteristic of marine origin, while the latter showed a terrestrial composition. From our previous studies (Mellbrand & Hambäck, 2010), we had found that chironomids generally have a marine carbon isotope signature, and the SIA would then suggest that the wolf spiders on reedy shores do not feed on chironomids. This conclusion contrasted with the molecular gut content analysis that indicated similar diets among spiders from open and reedy shores, at least when compared at the level of insect families. There was a variation in the specific prey species of spiders among sites, but the general prey groups (families/orders) where still very similar.

A comparison between the proportion of chironomids in the diet and the carbon isotope composition (Figure 3) offers a key to interpretation. On open shores, these two diet metrics were closely correlated, whereas on the reedy shores, there was no such relationship. Thus, the story on open shores seems straightforward, and the carbon isotope composition in spider legs reflects the proportion of chironomids in the diet. This result would appear if chironomids consumed by open shore spiders had a marine diet, such as live or dead green algae, while other prey groups had a more depleted carbon isotope signature. In this case, an increase in proportion of chironomids in the diet leads to a more marine signal in the spider. Recall that chironomids collected on open shores in the same region had δ^13^C = −18.4‰ (Enskog, 2006), which corresponds well with the estimated δ^13^C = −18.6‰ for a pure chironomid diet (Figure 2), while terrestrial prey found on these shores have δ^13^C < −25 (Mellbrand & Hambäck, 2010).

The story on reedy shores seems more complicated. The gut content analysis shows that spiders consumed chironomids also on these shores but this consumption did not seem to affect the carbon isotope composition of the spiders, as there was no relationship between δ^13^C and the proportion chironomids in the diet. However, if we assume that the molecular gut content analysis reflects the diet over the time when the carbon was incorporated in the spider legs, we can only conclude that the chironomids must have had terrestrial carbon in their body. Otherwise, we would have expected the carbon composition of spiders to vary with the proportion chironomids in the diet. Our presumption that eventual chironomids on the reedy shores would have a marine origin and a marine carbon isotope signal caused us not to specifically sample chironomids on these shores for isotope analysis, but this presumption in retrospect seems incorrect. However, even though we do not have access to stable isotope data for chironomids from these sites, we know that the spiders on reedy shores have a carbon isotope composition (δ^13^C = −26.2‰) resembling reed (δ^13^C = −26.6‰) and Diptera (brachycerid flies) found in reeds (δ^13^C = −25.6‰), but dissimilar to the isotope composition of other shoreline plants (δ^13^C = −30.3‰) (Enskog, 2006). If chironomids on the reedy shores feed on reed detritus, they would have δ^13^C similar to other Diptera feeding on live reed and it seems logical that the proportion chironomids in the diet should not correlate with δ^13^C.

When we examined the chironomid species in more detail, we detected no apparent difference in species composition among shore types, but the data are fairly sparse when resolved to this level, and several species of chironomids may feed on various types of detritus. There was, however, a tendency toward genera not occurring in the Baltic Sea, such as *Limnophyes* and *Metrocnemis* (Yngve Brodin, pers. comm.), being mainly present in spider guts from reedy shores. A difference in chironomid diets among shore types could also explain the outlier observed from one open shore site. On this site, spiders showed a terrestrial carbon isotope composition while still having chironomids in their gut—a pattern closely similar to that of spiders on reedy shores. Our interpretation is that these spiders had also fed on chironomids with a more terrestrial diet.

In either case, our analysis shows the limitations of stable isotope analyses. In cases where aquatic prey fed on terrestrial carbon, the resolution of SIA was insufficient to reveal the pathways for aquatic arthropods into the terrestrial food web. Moreover, it is possible and indeed even likely that not only chironomids but also other prey groups link spiders to the marine system. Of groups abundantly represented in spider guts, families Sphaeroceridae, Ephydridae, and Ceratopogonidae also feed on detritus either in the water or on shore deposits. While the life histories of these species are largely unknown, their potential roles as mediators of aquatic subsidies deserve further attention. Another abundant group among spider prey was Dolichopodidae. All species in this dipteran family are predatory both as larvae and adults (Oosterbroek, 2006). Among their main prey are various small dipterans potentially including chironomids, thus providing another link between spiders and the marine system.

The breadth of prey species found in the spider guts was astonishing. Among the 224 predation events observed in this study, we identified 105 different prey taxa. The true number of prey taxa was likely even higher, as some sequences were only identified to family or genus. While our study was not designed to test spider preferences for specific prey, the observed diversity of prey seems to suggest that the two wolf spiders in this study are largely opportunistic foragers, feeding on any dipteran or lepidopteran prey that are passing by, limited perhaps only by prey size. Nonetheless, opportunistic foraging does not imply nonselective foraging, only that selectivity is not caused by spider preference among prey. Specific prey taxa can still be over‐represented in the diet, when predator and prey activities overlap. For instance, *P. amentata* and *P. prativaga* typically hunt close to the ground (pers. obs.), and thus rarely catch prey in higher vegetation strata. High prey diversity would potentially contrast with the observation that spiders select prey based on nutrient content (e.g., Greenstone, 1979). The ability of individual wolf spiders to differentiate the quality of that many prey species in a field situation may limit their ability to forage for nutritional balance (due to similar neural limitations as in other arthropods, Bernays, 2001). Our findings may hopefully trigger novel research on foraging strategies by spiders when presented with a high diversity of prey taxa.

The two spider species included in the study apparently had different distribution. While *P. prativaga* occurred on both open and reedy shores, *P. amentata* seem to occur on only open shores. Even though we cannot deduce the cause for the different distributions, we can note that *P. prativaga* on open shores had a slightly more terrestrial carbon isotope signal than *P. amentata*, even though this difference was not apparent in the gut content analysis. Thus, it is possible that *P. amentata* is less able to catch prey in reedy habitats that have a more complex structure.

To conclude, we used complementary information from stable isotope analysis and molecular gut content analysis to provide further information on spider diets on shorelines, and on how the marine and terrestrial systems are linked. From the results, it is evident that either method alone would have provided an incomplete description of spider diets. SIA would have revealed only that spiders from open and reedy shores had different amount of marine carbon in their bodies, and molecular gut content analysis only that spiders from open and reedy shores had a similar taxonomic composition of their diets. Only together did the two analyses show a more complete picture. The strong correlation detected between the proportion of chironomids in spider guts and the stable isotope composition also suggests that molecular gut content analysis may provide quantitative insights into diet. By scoring independent predation events, and not relying on sequence counts from parallel sequencing, the proportional contribution of different prey items can apparently be fairly well quantified. In the specific case of spiders, this method is perhaps aided by the fact that a single prey item will typically dominate the gut contents of a sampled spider individual (cf. Wirta et al., 2015b). These tools may then allow us to place spiders in their proper place within food webs even when not leaving prey remains in their own web, and to identify the role of wolf spiders as vehicles of subsidies among habitats.

## Conflict of Interest

None declared.
