# Supplementary material for: Spatial subsidies in spider diets vary with shoreline structure: Complementary evidence from molecular diet analysis and stable isotopes
Source: Ecol Evol. 2016 Oct 26;6(23):8431–9. doi: 10.1002/ece3.2536 (PMC5167037; doi:10.1002/ece3.2536)
Supplement: Supplementary file 1 [file ECE3-6-8431-s001.docx]

Appendix S1. List of taxa identified from spider guts, and predation events recorded by Sanger and parallel sequencing respectively. Species names in bold were also collected in pan traps during the study, and were sequenced within the project.

|  |  | Predation events | |
| --- | --- | --- | --- |
| Insect family | Species identified in BOLD | Sanger | NGS |
| Chrysomelidae | *Chrysolina staphylea* | 0 | 1 |
| Chrysomelidae | *Galerucella tenella* | 0 | 1 |
| Staphylinidae | *Gabrius breviventer* | 0 | 1 |
| Agromyzidae | *Phytomyza n* sp. tenella | 1 | 0 |
| Anthomyidae | *Delia* sp | 0 | 1 |
| Anthomyidae | *Fucellia maritima/tergina* | 0 | 1 |
| Anthomyidae | *Zaphne frontata* | 0 | 1 |
| Anthomyidae | Unidentified species | 1 | 0 |
| Calliphoridae | *Lucilia sericata* | 0 | 1 |
| Calliphoridae | ***Melinda viridicyanea*** | 0 | 2 |
| Calliphoridae | *Morinia doronici* | 0 | 1 |
| Ceratopogonidae | *Atrichopogon forcipatus* | 1 | 0 |
| Ceratopogonidae | *Bezzia* sp 1 | 3 | 6 |
| Ceratopogonidae | ***Dasyhelea lucida*** | 4 | 0 |
| Ceratopogonidae | *Dasyhelea* sp1 | 1 | 1 |
| Ceratopogonidae | *Dasyhelea* sp9 | 2 | 1 |
| Ceratopogonidae | *Forcipomyia hygrophila* | 0 | 1 |
| Ceratopogonidae | *Forcipomyia* sp 2ES | 1 | 0 |
| Ceratopogonidae | *Palpomyia lineata* | 1 | 0 |
| Ceratopogonidae | Unidentified species | 2 | 3 |
| Chironomidae | *Chironomus aprilinus* | 1 | 0 |
| Chironomidae | *Cladotanytarsus atridorsum* | 1 | 3 |
| Chironomidae | *Cladotanytarsus mancus* | 5 | 4 |
| Chironomidae | *Cladotanytarsus pallidus* | 0 | 1 |
| Chironomidae | *Halocladius variabilis* | 1 | 0 |
| Chironomidae | ***Limnophyes asquamatus*** | 1 | 1 |
| Chironomidae | *Limnophyes pentaplastus* | 0 | 2 |
| Chironomidae | *Metriocnemus albolineatus* | 0 | 1 |
| Chironomidae | *Paratanytarsus dissimilis* | 3 | 0 |
| Chironomidae | ***Paratanytarsus natvigi*** | 9 | 4 |
| Chironomidae | *Paratanytarus* sp A | 1 | 5 |
| Chironomidae | *Paratendipes albimanus* | 0 | 1 |
| Chironomidae | *Polypedilum simulans* | 1 | 1 |
| Chironomidae | *Polypedilum uncinatum* | 1 | 0 |
| Chironomidae | *Polypedilum* sp9 SC | 1 | 1 |
| Chironomidae | *Pseudorthocladius* | 0 | 1 |
| Chironomidae | *Smittia* sp 8ES | 0 | 2 |
| Chironomidae | *Smittia edwardsi* | 1 | 0 |
| Chironomidae | *Stictochironomus maculipennis* | 1 | 1 |
| Chironomidae | *Tanytarsus desertor* | 0 | 2 |
| Chironomidae | *Tanytarsus usmaensis* | 0 | 1 |
| Chironomidae | Unidentified species | 0 | 3 |
| Chloropidae | ***Aphanotrigonum trilineatum*** | 1 | 1 |
| Chloropidae | *Cetema neglectum/myopinum* | 0 | 1 |
| Chloropidae | *Chlorops hypostigma* | 1 | 1 |
| Chloropidae | *Elachiptera tuburculifera* | 0 | 1 |
| Chloropidae | *Oscinella* sp 4 | 0 | 2 |
| Culicidae | *Aedes leucomelas* | 0 | 1 |
| Culicidae | *Culicoides newsteadi* | 0 | 2 |
| Culicidae | *Ochlerotatus sticticus* | 0 | 1 |
| Culicidae | *Ochloretatus/Aedes sp A* | 1 | 0 |
| Dolichopodidae | *Dolichopus nubilus/austriacus* | 12 | 2 |
| Dolichopodidae | ***Dolichopus longitarsis*** | 3 | 2 |
| Dolichopodidae | *Dolichopus plumipes/simplex* | 2 | 1 |
| Dolichopodidae | *Dolichopus sabinus* | 1 | 2 |
| Dolichopodidae | *Dolichopus ungulatus* | 4 | 6 |
| Dolichopodidae | *Sympycnus pulicarius* | 0 | 1 |
| Drosophilidae | ***Scaptomyza pallida*** | 3 | 1 |
| Empididae | *Rhamphomyia albipennis/murina* | 1 | 0 |
| Empididae | *Rhamphomyia geniculata* | 0 | 1 |
| Ephydridae | *Lamproscatella sibilans* | 1 | 0 |
| Ephydridae | ***Scatella tenuicosta*** | 12 | 8 |
| Hybotidae | *Platypalpus cursitans* | 0 | 1 |
| Hybotidae | *Bicellaria spuria/simplicipes/sulcata* | 1 | 4 |
| Hybotidae | *Stilpon graminum* | 0 | 1 |
| Limoniidae | *Dicranomyia autumnalis* | 2 | 1 |
| Limoniidae | *Gonomyia tenella* | 1 | 1 |
| Limoniidae | *Orimarga juvenilis* | 0 | 1 |
| Lonchopteridae | *Lonchoptera lutea* | 1 | 0 |
| Muscidae | *Coenosia pumila/perpusilla/palludis* | 1 | 0 |
| Muscidae | *Drymeia segnis ** | 0 | 1 |
| Muscidae | *Phaonia bidentata ** | 0 | 1 |
| Muscidae | *Spilogona megastoma* | 0 | 1 |
| Phoridae | *Megaselia pleuralis* | 1 | 1 |
| Phoridae | *Megaselia sp. AK-2010-2* | 1 | 1 |
| Phoridae | Unidentified species | 1 | 0 |
| Sarcophagidae | *Sarcophaga depressifrons* | 1 | 1 |
| Scatopsidae | ***Thripomorpha verralli*** | 1 | 1 |
| Scatophagidae | *Spaziphora hydromyzina* | 1 | 0 |
| Sciomyzidae | *Limnia paludicola/unguicornis* | 0 | 2 |
| Sphaeroceridae | *Leptocera fontinalis* | 1 | 4 |
| Sphaeroceridae | ***Opacifrons coxata*** | 2 | 2 |
| Sphaeroceridae | *Pullimosina pullula* | 0 | 1 |
| Sphaeroceridae | ***Rachispoda lutosa*** | 3 | 1 |
| Sphaeroceridae | *Spelobia* sp A | 11 | 4 |
| Sphaeroceridae | Unidentified species | 4 | 0 |
| Syrphidae | *Baccha elongata* | 0 | 1 |
| Tabanidae | *Haematopota subcylindrica/pluvialis* | 0 | 1 |
| Ulidiidae | *Coroxys urticae* | 1 | 0 |
| Diptera | Unidentified species | 2 | 2 |
| Miridae | *Stenodema trispinosa* | 1 | 1 |
| Miridae | *Lygus* sp A (7 species) | 1 | 0 |
| Coleophoridae | *Coleophora glaucicolella* | 1 | 1 |
| Crambidae | *Acentria ephemerella* | 0 | 1 |
| Elachistidae | *Elachista eleochariella* | 0 | 1 |
| Glechidae | *Scrobipalpa obsoleta* | 1 | 0 |
| Geometridae | *Petrophora chlorosata* | 1 | 1 |
| Momphidae | *Mompha langiella* | 1 | 0 |
| Noctuidae | *Deltote uncula* | 1 | 0 |
| Pieridae | *Colias palaeno* | 0 | 1 |
| Tortricidae | *Celypha lacunana* | 1 | 1 |
| Tortricidae | *Cnephasia asseclana* | 0 | 1 |
| Tortricidae | *Olethreutes* sp A | 0 | 1 |
| Tortricidae | *Bactra robustana* | 2 | 1 |
| Tortricidae | *Bactra lacteana* | 0 | 1 |

* species not recorded in Sweden
